# Laccase bound to cryogel functionalized with phenylalanine for the decolorization of textile dyes

**DOI:** 10.3906/kim-2106-16

**Published:** 2021-10-19

**Authors:** Işık PERÇİN, Yusuf Doruk ARACAGÖK, Neslihan İDİL, Adil DENİZLİ, Bo MATTIASSON

**Affiliations:** 1 Department of Biology, Molecular Biology Division, Faculty of Science, Hacettepe University, Ankara Turkey; 2 Department of Biology, Biotechnology Division, Faculty of Science, Hacettepe University, Ankara Turkey; 3 Department of Chemistry, Biochemistry Division, Faculty of Science, Hacettepe University, Ankara Turkey; 4 Division of Biotechnology, Lund University, Lund Sweden

**Keywords:** Laccase, N-methacryloyl-(L)-phenylalanine methyl ester, cryogel, textile dye decolorization, reactive blue

## Abstract

In this study, amino acid functionalized poly(2-hydroxyethyl methacrylate-N-methacrylolyl-l-phenylalanine) [PHEMAPA] cryogel discs were prepared. In this respect, phenylalanine containing N-methacryloyl-(L)-phenylalanine methyl ester (MAPA) was polymerized with 2-hydroxyethyl methacrylate (HEMA) without requirement of any activation step. Laccase bound poly(2-hydroxyethyl methacrylate-N-methacryloyl-l-phenylalanine) [Lac-PHEMAPA] cryogel discs were applied for decolorization of Reactive Blue-247 (RB-247). The ability of Lac-PHEMAPA cryogel discs on dye decolorization was found to be as 90% in 2 h and even more within 4h. The decolorization activities of 86% and 73% were observed at relatively low (4°C) and high (60°C) temperatures, respectively. The effect of dye concentration on dye decolorization and 100% decolorization activity was achieved in dye concentration between 50–300 ppm. Lac-PHEMAPA cryogel discs maintained 80% of its decolorization activity after six cycles. Consequently, the PHEMAPA cryogel discs are promising materials for immobilizing laccase. The Lac-PHEMAPA has a rapid dye decolorization in a broad range of temperature. The preparation is furthermore very stable and activity is preserved during storage.

## 1. Introduction

Azo dyes constitute a group of dye stuffs, which have gained great commercial interest in the textile industry. However, the excess amount of these dyes is released as effluent wastewater. Undesirable dyeing agents present in water lead to critical environmental problems and also effects on public health due to their toxic, mutagenic, and carcinogenic features [1]. Although some traditional physicochemical methods have been applied to treat wastewater by removal of colour in various steps, these techniques have many limitations such as the large quantity of chemicals consumed, incapability of fully dye removal, formation of toxic metabolites, production of a sludge, which contains also other pollutants which needs complex multistep methodologies for efficient treatment [2]. Degradation of Azo dyes involves elimination of the colour and then also to degrade the metabolites. Therefore, it is necessary to implement several techniques, because using one of them alone can not meet all requirements for effective dye decolorization and degradation of metabolites [3]. Nevertheless, decolorization of these dyes using microbial enzymes represents an eco-friendly and relatively inexpensive option over the other degradation processes. Furthermore, use of enzymes minimize the water consumption. Oxidases is a group among the enzymes, which plays an important role in the decolourization. Especially laccases have been extensively used due to their widespread availability and have been used in decolorization of several dyes. Laccase (EC 1.10.3.2) presents the polyphenol oxidases and oxygen is reduced to water with the enzymes’ catalytic activity. This enzyme has the ability of oxidizing many phenolic compounds and aromatic amines without any requirement of additives [4]. Laccase in combination with mediators constitute an effective combination when used in e.g., membrane reactors [5]. The abundance of laccase in different white-rot fungi species and production and purification of the enzyme from stable microbial sources have already been preferred in many commercial applications [6]. These properties make this enzyme unique in the textile dyeing industry. Besides, there is a growing need in the development of different approaches in dye decolorization using enzymes. In recent years, great attention has been given to immobilization processes, which provide improved reusability and stability, cost-effective system generation [7–11]. For this process, cryogels are popular polymeric materials offering a promising future with their supermacroporous structure. They are easily prepared at freezing temperature, and large pores provide some advantages such as low pressure drop, efficient diffusion property and short residence time. These characteristics of cryogels make them unique and applicable to be preferred in many fields such as medical [13, 14] environmental [15–17] and biotechnological applications [18, 19]. Poly(2-hydroxyethyl methacrylate) [PHEMA] based support materials have the extensive potential for several applications, including enzyme immobilization because of their improved performance at a large scale of different experimental conditions [20, 21]. These materials can also be functionalized by novel methodologies, in this way, the hydrophilic character of PHEMA may be modified via using different monomers and ligands [22]. MAPA is a phenylalanine containing aminoacid-derived functional monomer. It is used to gain hydrophobic functionality to the support material and can interact with glycoproteins such as laccase. Therefore, the work presented herein indicates the binding mechanism of MAPA and laccase that can be attributed to the interaction between hydrophobic groups of MAPA and laccase [23]. 

In this study, laccase bound to cryo-polymerized support material was aimed to be used for dye decolorization - a challange that needs to be met in textile industrial scale applications. For this purpose, we have directed our attention to examine optimized conditions for ability of Lac-PHEMAPA cryogel discs. Lac-PHEMAPA cryogel discs were prepared, and efficiency in decolorization was studied. Effects of reaction time, pH value, temperature and dye concentration on decolorization efficiency of Lac-PHEMAPA cryogel discs were investigated. Besides, storage stability and reusability of Lac-PHEMAPA cryogel discs were determined. 

## 2. Materials and methods

### 2.1. Materials 

Laccase from *Trametes versicolor*, 2,2’- azino-bis(3-ethylbenz-thiazoline-6-sulphonic acid) (ABTS), 2-hydroxyethyl methacrylate (HEMA), methylenebisacrylamide (MBAA), *N,N,N’,N’*-tetra-methyl-ethylenediamine (TEMED), and ammonium persulphate (APS) were obtained from Sigma-Aldrich (SigmaChem, USA). RB-247 was obtained from the textile industry. All other chemicals used were of analytical grade. All buffers were prepared with water processed using a reverse osmosis step with a Milli-Q system from Millipore (Bedford, MA, USA). 

### 2.2. Synthesis of MAPA monomer

The experimental procedure applied for the synthesis of MAPA monomer was summarized in the following as reported elsewhere [24]. (L)-phenyl alanine methyl ester (C_6_H_5_CH_2_CH(NH_2_)COOCH_3_; 5.0 g) and sodium nitrite (NaNO_2_; 0.2 g) were dissolved in 30 mL water containing 5% w/v potassium carbonate (K_2_CO_3_). The reaction chamber was cooled to 0 °C in an ice bath and the mixture was stirred magnetically under a nitrogen atmosphere. Then, methacryloyl chloride (C_4_H_5_ClO; 4.0 mL) was added slowly into a reaction solution. The obtained solution was stirred magnetically at 100 rpm and 24 °C for 2 h. The product was extracted with ethyl acetate and the aqueous phase was evaporated. The residue was crystallized to obtain MAPA monomer. 

### 2.3. Preparation of PHEMAPA cryogel discs

PHEMAPA cryogel discs were prepared by cryopolymerization as described as follows. In the first step, 1.3 mL of HEMA and functional monomer MAPA (200 mg) were dissolved in 5 mL of H_2_O. In another beaker, 0.283 g of MBAA, as the crosslinker, was dissolved in 10 mL of H_2_O. Then, MBAA and monomer mixture were mixed until they are completely dissolved. The mixture was cooled on an ice bath. After adding APS and TEMED, the mixture was poured between two glass plates using spacers 1.5 mm in thickness and was put in deep freeze at –18°C and was left for 24 h for polymerization to take place. The formed cryogel was then thawed at room temperature, and the cryogel layer was cut into circular pieces to be 0.8 cm in diameter. PHEMAPA cryogel discs were washed several times by water and alcohol to remove unreacted monomers. PHEMAPA cryogel discs were stored in 0.02% sodium azide solution until they are used. 

### 2.4. Characterization of PHEMAPA cryogel discs 

To determine polymerization yield, PHEMAPA cryogel discs were dried at vacuum oven and weighed. Eq.1 was used to calculate yield: 

*Yield (%) = (m* *_dried gel_* */ m* *_t_* *) 100 *(1) 

Here, m_dried gel _represents the mass of dried cryogel disc; m_t _represents the total mass of monomers. 

Swelling degrees of PHEMAPA cryogel discs were calculated according to Eq. 2. Firstly, they were kept in water until they were completely swollen. Then, they were placed on dried paper and excess water was removed. Weights of PHEMAPA cryogel discs (m_wet gel) _were determined. Then, cryogel discs were dried at 55°C and weighted (m_dried gel_). 

*Swelling degree (S) = (m* *_wet gel_* *- m* *_driedgel_* *) / m* *_dried gel_* (2) 

To determine macroporosity, the weights of the swollen cryogels were determined (m_swollen gel_). Then, water was removed from swollen cryogels by squeezing, and the weights of the squeezed cryogels (m_squeezed gel_) were determined. Eq. 3 was used to calculate macroporosity. 

*Macroporosity (%) =(m* *_swollen gel_* *– m* *_squeezed gel_* *) /m* *_swollen gel_* *x 100* (3)

PHEMAPA cryogel discs were characterized by FTIR (Thermo Fisher Scientific, Nicolet iS10, Waltham, MA, USA) to determine the presence of phenylalanine. The samples were dried at 55 °C before analysis. 

Surface morphology of PHEMAPA cryogel discs was examined by SEM (JEOL, JEM 1200 EX, Tokyo, Japan). Firstly, PHEMAPA cryogel discs were frozen at –20 °C and completely freeze dried in a lyophilizer (Chris Alpha 1-2 LD Freeze Dryer, SciQuip, England). Then, the samples were coated with metallic gold, and SEM photos of them were taken in different magnifications. 

### 2.5. Laccase binding studies

Binding of laccase on PHEMAPA cryogel discs was performed in a batch experimental set-up. PHEMAPA cryogel discs were equilibrated with 50 mM, pH = 6.0 phosphate buffer for 2h. Laccase solutions were prepared by dissolving laccase in 15 mL of 50 mM, pH = 6.0 phosphate buffer, and final concentrations of laccase solutions were changed between 0.1 and 1.5 mg/mL. Laccase binding experiments were performed at 25°C for 18 h. After laccase binding process, Lac-PHEMAPA cryogel discs were washed with 50 mM, pH = 6.0 phosphate buffer for three times to remove excess free enzyme molecules. Also, effect of pH value (5.0–8.0) on binding of laccase on PHEMAPA cryogel discs was investigated. 

Bound amount of laccase was determined according to Eq. 4 by measuring the initial and final laccase concentration spectrophotometrically at 650 nm (Shimadzu, Japan) by Lowry method [25].

*Q = (C* *_i_* *-C* *_f_* *)V/m* (4)

Here, Q is the amount of bound laccase on a unit mass of PHEMAPA cryogel discs (mg/g), C_i_ is the initial concentration of laccase (mg/mL), C_f_ is the equilibrium concentration of laccase after binding (mg/mL), V is the volume of aqueous binding solution (mL), and m is the mass of dried cryogel discs used (g).

### 2.6. Dye decolorization

Dye decolorization ability of Lac-PHEMAPA cryogel discs was determined with the pigment RB-247 in a batch system. RB-247 was dissolved in distilled water. The final concentration of the dye solution was 50 mg/L. Three pieces of Lac-PHEMAPA cryogel discs (21 mg) were added to the dye solution, and dye decolorization process was performed at 25 °C for 8 h. Decolorization efficiency of Lac-PHEMAPA cryogel discs was determined by measuring solutions of RB-247 (before and after decolorization) spectrophotometrically at 612 nm (Shimadzu UV-1700). The decolorization yield was calculated according to Eq. 5;

Decolorization (%) = (Initial Absorbance-Final Absorbance)/(Initial Absorbance)*100 (5)

Effect of pH on dye decolorization ability of Lac-PHEMAPA cryogel discs was investigated. Initial pH of the dye solution was changed between 3.0 and 8.0. Besides, the effect of temperature on dye decolorization ability of Lac-PHEMAPA cryogel discs was examined at different temperatures (4, 10, 20, 30, 40, 50, 60 °C). On the other hand, Lac-PHEMAPA cryogel discs were stored at 4 °C for three months to examine storage stability of Lac-PHEMAPA cryogel discs. Maintaining of dye decolorization capability of Lac-PHEMAPA cryogel discs was evaluated after three months of storage. 

Usage of cheap and reusable enzyme bound to materials in the industry for dye decolorization processes are important. Therefore, same Lac-PHEMAPA cryogel discs were used ten times in decolorization process and decolorization efficiency of Lac-PHEMAPA cryogel discs was tested in each step. No dye desorption process was applied between decolorization steps.

## 3. Results and discussions

**Figure 1:**
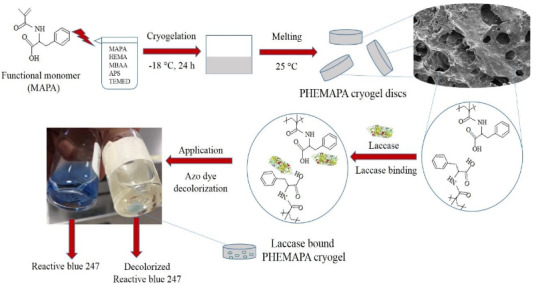
The schematic presentation of the dye decolorization process using Lac-PHEMAPA cryogel discs.

Materials with immobilized laccase have been applied for the treatment of textile wastewater pollutant. In literature, there have been many publications indicating applications of laccase on dye decolorization [26-30]. In most of these studies, the superiority of immobilized laccase over free laccase, and the ability of immobilized laccase on dye decolorization was reported. Apart from these studies, application of laccase in the industrial scale has been taken into consideration and for this purpose optimum conditions of dye decolorization were investigated in our study. The schematic presentation of the overall methodology and dye decolorization process is briefly illustrated in Figure 1.

Textile wastewater shows markedly fluctuating pH and temperature values [31]. In addition to these parameters, initial dye concentration and reaction time have significant effects on dye decolorization [32]. By this reason, our paper focuses on examining the efficiency of Lac-PHEMAPA cryogel discs on dye decolorization with confirming their properties on enzyme stability and reusability of Lac-PHEMAPA cryogel discs. 

### 3.1. Characterization studies

**Figure 2:**
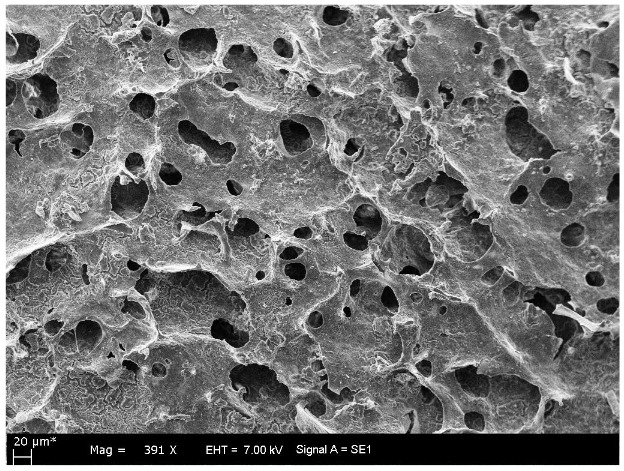
SEM image of PHEMAPA cryogel discs.

SEM images of PHEMAPA cryogel discs are given in Figure 2. Unique characteristics of cryogels can be clearly seen on SEM images of the PHEMAPA cryogel discs. Bulk structure of the PHEMAPA cryogel discs was characterized with interconnected large pores. Some swelling properties of PHEMA and PHEMAPA cryogel discs are also given in Table 1. Macroporous structure of PHEMAPA cryogel discs provided the high swelling ratio (987%) and high swelling degree (9.8 g_H2O_/g_polymer_) values for PHEMAPA cryogel discs. Furthermore, macroporosity of the PHEMAPA cryogel discs was found as 76.7% and gelation yield was obtained as 89%. Thus, monomers HEMA and MAPA were mostly polymerized to get the whole structure of the PHEMAPA cryogel. 

**Table 1:** Characteristics of PHEMA and PHEMAPA cryogel discs.

	Swelling ratio(%)	Swelling degree(gH2O/gpolymer)	Macroporosity(%)	Gelation yield (%)
PHEMA cryogel discs	889	8.9	75	90
PHEMAPA cryogel discs	987	9.8	76.7	89

**Figure 3:**
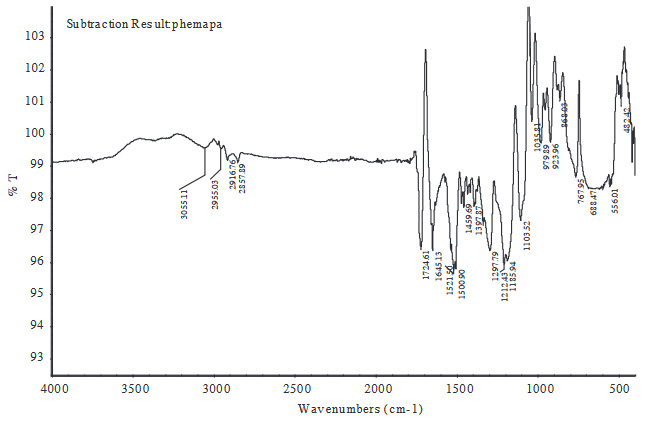
The subtraction of FTIR spectrum of PHEMAPA cryogel discs.

FTIR spectrum analysis of PHEMA and PHEMAPA cryogel discs were performed to show the incorporation of MAPA within the cryogel structure. The subtraction result of FTIR spectrum of PHEMA from PHEMAPA is shown in Figure 3. Characteristic stretching vibration band of ester around 1645 and 1521 cm^–1^, aromatic C-H banding around 1297, 1212 and 3055 cm^–1^, additional ester around 1724 indicated the incorporation of MAPA functional monomer in cryogel structure.

### 3.2. Laccase binding studies

#### 3.2.1. Effect of pH value

Determination of optimum pH has a significant influence on enzyme binding. The effect of pH on laccase binding on PHEMAPA cryogel discs is given in Figure 4a. Maximum laccase binding on PHEMAPA cryogel discs was obtained at pH 6.0. Laccase binding capacity of PHEMAPA cryogel discs was decreased at pH values lower and higher than pH 6.0. MAPA as a functional monomer has a double mode of action providing aromatic and hydrophobic interactions together. Binding between PHEMAPA cryogel discs and laccase molecules can be attributed mostly to hydrophobic and aromatic interactions. Laccase is a complex glycoprotein [33]. Hydrophobic amino acids and saccharide groups of laccase molecules are expected to interact with phenylalanine groups on PHEMAPA cryogel discs [34, 35].

**Figure 4a:**
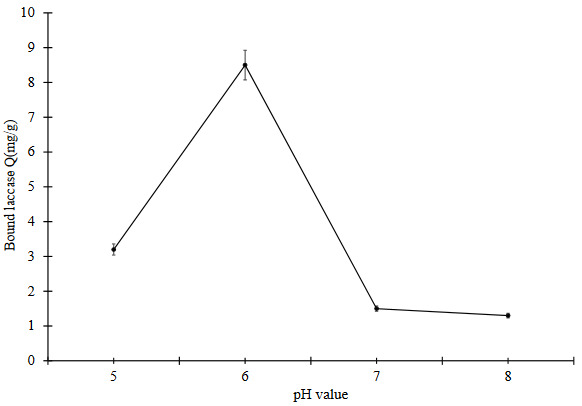
Effect of pH value on laccase binding on PHEMAPA cryogel discs. Initial concentration of laccase: 0.1 mg/mL, temperature: 25 °C. All data were reported as mean ± SD, n = 3.

**Figure 4b:**
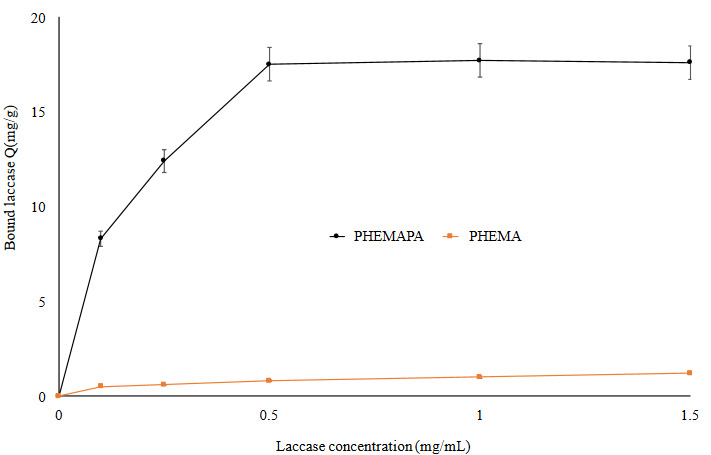
Effect of initial laccase concentration on laccase binding on PHEMAPA cryogel discs. Binding buffer: pH = 6.0 phosphate buffer, temperature: 25 °C. All data were reported as mean ± SD, n = 3.

#### 3.2.2. Effect of the initial concentration of laccase

The effect of initial laccase concentration on laccase binding capacity of PHEMAPA cryogel discs is presented in Figure 4b. The maximum amount of laccase bound on PHEMAPA cryogel discs was 17.5 mg laccase/g cryogel. As it can be seen from the figure, after 0.5 mg/mL of laccase there is no meaning increase the amount of bound laccase. Therefore, it is not economical to load more laccase per unit of PHEMAPA cryogel discs. Thus, we used Lac-PHEMAPA cryogel discs carrying 17.5 mg/g laccase in other experimental studies. Laccase binding capacity of PHEMA cryogel discs is also shown in Figure 4b. Laccase binding on PHEMA cryogel discs was very low. As a result, laccase binding on PHEMAPA cryogel discs occurs via specific interaction between MAPA groups and laccase molecules. 

Modelling of the equilibrium data was applied using Langmuir and Freundlich isotherms [36]. Langmuir adsorption isotherm was defined in Eq.6 

*Q = Q* *_max_* *. b. C* *_eq_* */ (1 + bC* *_eq_* *)* (6)

Here, Q is the amount of bound laccase on PHEMAPA cryogel discs(mg/g), C_eq_ is the equilibrium concentration of laccase (mg/mL), b is the Langmuir constant (g/m_mol_) and Q_max_ is the binding capacity of PHEMAPA cryogel discs. Freundlich isotherm was defined in Eq.7

*Q* *_e_* *= K* *_f_* *C* *_e_* *^1/n^* (7)

Langmuir and Freundlich adsorption isotherms were given in Table 2. According to the results, R^2^ value for Langmuir adsorption isotherm is higher than the R^2^ value for Freundlich isotherm. Thus, our system fits Langmuir isotherm and laccase molecules were placed on PHEMAPA cryogel discs as a single layer. The obtained results indicated that there was no steric hindrance when laccase molecules interacted with special binding sites on PHEMAPA cryogel discs. Furthermore, equal energy binding, homogeneous interaction sites and no lateral interaction which are related to Langmuir adsorption are valid for our affinity binding system.

**Table 2:** Langmuir and Freundlich binding isotherm constants for laccase binding on PHEMAPA cryogel discs.

	Experimental	Langmuir constants			Freundlich constants		
	Q (mg/g)	Qmax(mg/g)	b (mL/mg)	R2	Qf	n	R2
PHEMAPA cryogel discs	17.50	19.72	8.89	0.97	19.2	3.14	0.94

First- and second-order kinetic models were applied to the experimental data for determining mechanisms controlling adsorption process like mass transfer and chemical reaction [37]. The pseudo first order kinetic of Lagergren is the most widely used equation for the adsorption of solute from a liquid solution, and it is defined in Eq. 8.

*log[q* *_eq_* */ (q* *_eq_* *– q* *_t_* *)] = (k* *_1_* *t) / 2.303 *(8)

Here, q_eq_ is the experimental amount of laccase bound at equilibrium (mg/g), q_t_ is the amount of laccase bound at time t (mg/g) and k_1_ is the rate constant.

The pseudo second order kinetic model is defined in Eq. 9.

*(t/qt) =(1 / k* *_2_* *q* *_eq_* *^2^* *) +(1/ q* *_eq_* *) t* (9)

Here, k_2_ is the rate constant of the pseudo-second-order adsorption (g mg^–1^min^–1^). If the pseudo-second-order kinetics is applicable, the plot of t/q versus t should be linear.

Pseudo-first and -second order kinetic models for binding of laccase on PHEMAPA cryogel discs are summarized in Table 3. When the results were evaluated, the correlation coefficient was higher for pseudo second order kinetics. Therefore, the affinity system in this study could be explained by pseudo-second order kinetics. The binding of laccase on PHEMAPA cryogel discs did not affect diffusion limitations. Macropores inside PHEMAPA cryogel structure prevented from flow resistance. As a result, specific interactions between laccase and phenylalanine controlled kinetic behaviour. In other words, binding of laccase on PHEMAPA cryogel discs was chemically controlled and was performed without diffusion limitations.

**Table 3:** The first- and second-order kinetic constants.

Equilibrium concentration	Experimental	Pseudo-first order			Pseudo-second order		
	Qeq (mg/g)	k1(1/min)	qeq(mg/g)	R2	k2(g/mg.min)	qeq(mg/g)	R2
0.5 mg/mL	17.50	0.02	3.34	0.28	0.01	17.73	0.99

### 3.3. Dye decolorization using Lac-PHEMAPA cryogel discs

#### 3.3.1. Effect of reaction time on dye decolorization

**Figure 5:**
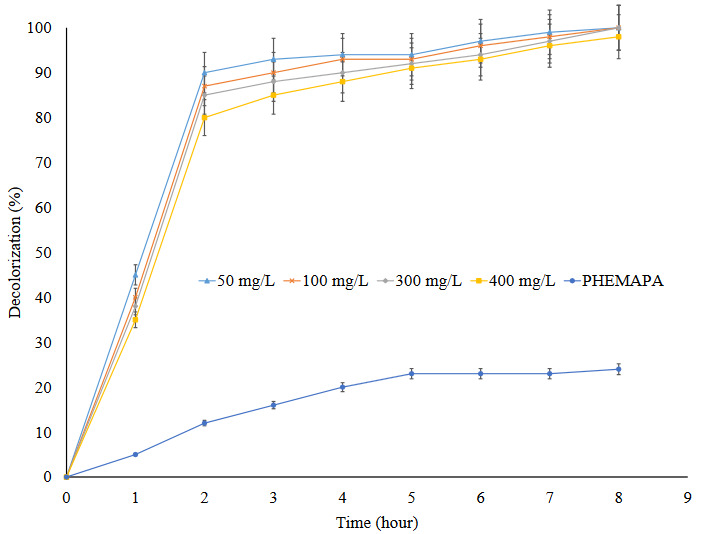
Effect of time on dye decolorization efficiency of Lac-PHEMAPA cryogel discs. Temperature: 25 °C. All data were reported as mean ± SD, n = 3.

Effect of reaction time on dye decolorization efficiency of Lac-PHEMAPA cryogel discs was given in Figure 5. As can be clearly seen from the figure, the ability of Lac-PHEMAPA cryogel discs on dye decolorization was found to be as 90% in 2 h. It was observed that Lac-PHEMAPA cryogel discs were capable to decolorize the dye RB-247 with 100% efficiency in 4 h. 

Furthermore, PHEMAPA cryogel discs and Lac-PHEMAPA cryogel discs were compared in terms of dye decolorization capabilities in the same conditions as stated above. Obtained results are given in Figure 5. According to the figure, it is clear that Lac-PHEMAPA cryogel discs were able to decolorize RB-247 with a high percentage (almost 100%), whereas PHEMAPA cryogel discs were capable of absorbing 24% of RB-247 dye. Therefore, obtained decolorization efficiency of Lac-PHEMAPA cryogel discs on RB-247 can be attributed to biodegradation ability of cryogel discs.

#### 3.3.2.Effect of medium pH on dye decolorization

**Figure 6:**
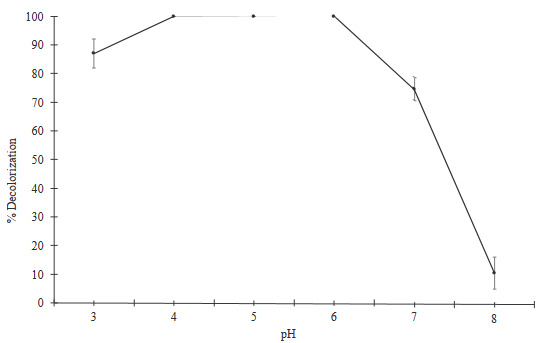
Effect of pH value on dye decolorization efficiency of Lac-PHEMAPA cryogel discs. Temperature: 25 °C. Initial RB-247 concentration: 50 mg/L. All data were reported as mean ± SD, n = 3.

Effect of pH on dye decolorization of Lac-PHEMAPA cryogel discs was investigated and obtained results are shown in Figure 6. As clearly seen in the figure, Lac-PHEMAPA cryogel discs showed maximum dye decolorization activity (almost 100%) at the range of pH = 4–6. Also, dye decolorization activity decreased dramatically below pH 4.0 and above pH = 6.0, only 11% decolorization rate was observed at pH = 8. Different pH values have effect on dye decolorization efficiency. Therefore, pH adaptability of Lac-PHEMAPA cryogel discs in a wide range of pH values provides an opportunity for treatment of dye contaminated effluents. 

#### 3.3.3. Effect of temperature on dye decolorization

**Figure 7:**
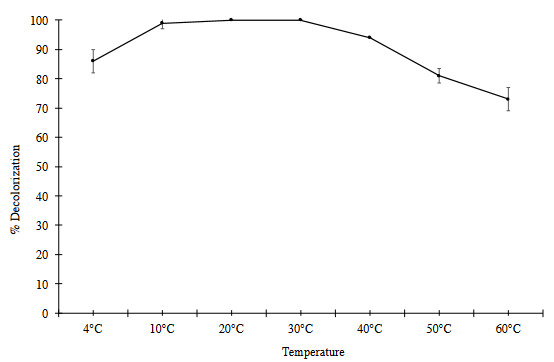
Effect of temperature on dye decolorization efficiency of Lac-PHEMAPA cryogel discs. Initial RB-247 concentration: 50 mg/L (pH 4.5). All data were reported as mean ± SD, n = 3.

Temperature is a critical parameter for enzyme stability. Effect of temperature on dye decolorization of Lac-PHEMAPA cryogel discs was investigated**. **Seven different temperatures (4, 10, 20, 30, 40, 50, 60 °C) were tested to determine the effect of temperature on dye decolorization and obtained results were presented in Figure 7. It can be seen that Lac-PHEMAPA cryogel discs showed 86% decolorization activity even at 4 °C. Also, dye decolorization efficiency of 100% was observed between 10–30 °C. Lac-PHEMAPA cryogel discs had the ability of 73% dye decolorization activity at relatively high temperature (60 °C). 

#### 3.3.4. Storage stability and reusability

To determine storage life of immobilized enzymes, Lac-PHEMAPA cryogel discs were stored at +4 °C for three months. After three months, Lac-PHEMAPA cryogel discs showed close to 100% decolorization efficiency. 

**Figure 8:**
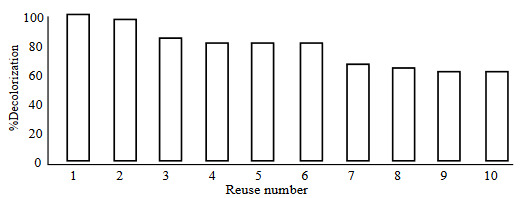
Reusability of Lac-PHEMAPA cryogel discs. Temperature: 25 °C. Initial RB-247 concentration: 50 mg/L (pH 4.5). All data were reported as mean ± SD, n = 3.

Reusability of Lac-PHEMAPA cryogel discs were tested for their decolorization efficiency, and obtained results are shown in Figure 8. Lac-PHEMAPA cryogel discs maintained 80% and 60% of its decolorization activity after six cycles and ten cycles, respectively. It is noteworthy to say that, the reusability experiments were performed without any dye desorption process between each dye decolorization cycle. 

## 4. Comparison with literature

There have been several studies in the literature aiming decolorization of textile dyes using different kinds of physical and chemical methods [38–40]. Even though these methods find a great range of availability and applicability for the decolorization of wastes contaminated with dye, optional assays relied on biotechnological methodologies has received great interest. Laccase immobilized on matrices have taken place as one of the most widely used materials for this purpose [27, 41–44]. It is noteworthy saying that immobilization of laccase enhances decolorization efficiency and cost-effectively provide sustainable approach with the advantages of reusability and long-term storage stability. 

Usually, up to recent days, polymeric support materials consisting of polyacrylamide and alginate were frequently preferred for enzyme immobilization. However, porous materials have been introduced as effective matrices for immobilization of enzymes with low cost and high mechanical stability. 

Decolorization ability of laccase on the decolorization of textile dyes has been reported in many types of research. One of these studies showed that laccase from *Trametes modesta* immobilized on γ-aluminum oxide pellets were applicable for the decolorization of anthrachinonic dyes (Lanaset Blue 2R, Terasil Pink 2GLA) and azo dyes, Indigo Carmine, and the triphenyl- methane dye Crystal Violet [45].

In a previous study, laccase immobilized poly(MA- alt-MVE)-g-PLA/ODA-MMT nanocomposite was prepared for the decolorization of Reactive Red 3. Effects of different experimental conditions (pH value, temperature, dye concentration and reaction time) were determined for optimizing the decolorization process. Decolorization efficiency of laccase (0.05 mg/mL laccase concentration) immobilized poly(MA-alt-MVE)-g-PLA/ODA-MMT nanocomposites was found as 65% in pH = 5.0 at 20 ºC for 90 min [46]**.**

In another study, laccase from *Tramates versicolor* immobilized on porous glass beads and their efficiency on dye decolorization were investigated. Anthraquinone (Reactive blue 19 and Dispersed blue 3), indigoid (Acid blue 74) dyes and azo dyes (Acid red 27 and Reactive black 5) were decolorized. The results from this study indicated that different decolorization rates were obtained for mentioned dyes defined as 40.6% for acid red 27, 12.0% for reactive black 5, 74.0% for acid blue 74, 78.0% for dispersed blue, 77.0 for reactive blue 19 [42].

A previous research reported that ZnO nanowires into macroporous SiO2 formed composite material used for laccase immobilization. The dye decolorization ability of resultant support material was examined. As a result of this study, 93% and 82% decolorization rates were reported for Remazol brilliant blue B, and acid blue 25, respectively. In accordance with the results of our study, laccase immobilized composite indicated enhanced thermal stability and pH adaptability. In addition, laccase immobilization leads to maintained 42% decolorization ability after ten cycles [47].

In a previous study, sol-gel synthesis of biopolymer-silica hybrids was preferred as matrices for laccase immobilization. They reported 84% Malachite green degradation ability for 72 h [26].

In another study, laccase trapped beads consisting of alginate/gelatin blend with polyethylene glycol was used for dye decolorization. Glutaraldehyde activated materials have enhanced decolorization ability. Reactive Red B-3BF (50 mg/L) was decolorized down to 50% after ten cycles [48].

Purified laccase from *Trametes hirsuta* decolorize effectively triarylmethane, indigoid, anthraquinonic and azo dyes [49]. Another study showed that immobilized laccase more than 70% of retained its activity after 5 months storage at +4 °C [44]. In a previous study in literature, laccase immobilized on Poly(MMA-co-GMA) cyogel showed maximum activity at pH = 4 and immobilized laccase activity decrease dramatically above and below pH = 4 [50]. Another study showed that *Trametes versicolor* crude laccase showed maximum activity at pH = 4.5 [51]. *Lentinus polychrous* crude laccase showed maximum Acid Blue 80 decolorization activity around pH = 5 [52]. In this study, immobilized laccase showed maximum dye decolorization activity at pH = 4, 5, 6 and decolorization rate achieved above 70%. 

Textile dyes are chosen for the cloths since the pigment is very robust and will be resistant to bleaching and degradation. The surplus dyes that are released from industries are, therefore, a challenge. Use of high concentrations of immobilized enzymes is one approach; another approach might be to combine, for example, laccase with nanoparticles of titanium, which will become an efficient partner to the laccase when exposed to UV-light, since titanium creates activated oxygen that is a potent reagent for degrading aromatic structures [52–55]. In order not to destroy the enzymes by oxidation from the activated oxygen from the titanium, one would need to use sequential treatment of the dyes in order to degrade the dye molecules and also to degrade the metabolites formed.

## 5. Conclusion

Dye effluents lead to important environmental problems. Therefore, coping with these effluents is of great concern and troublesome because of the complexity of dye contaminants. The techniques developed for this aim are cost effective and sophisticated. Consequently, easy to prepare and cheap decolorization techniques have gained attention to manage dye effluents. In this study, the ability of laccase bound to (PHEMAPA) cryogel discs was indicated for dye decolorization using a model dye, Reactive Blue 247. MAPA was preferred to eliminate the activation process for enzyme binding and cryogel discs known as novel polymeric systems were used because of their unique structural features and advantageous dynamics. After using a model substrate (ABTS) for determining laccase activity of laccase bound (PHEMAPA) cryogel discs, the experimental conditions were optimized in order to determine the best performance of immobilized laccase on (PHEMAPA) cryogel discs in dye decolorization. When the facility of these cryogels was taken into consideration, it could be suggested that these materials are promising tools for enzyme binding along with dye decolorization by the advantage of excellent enzyme activity in a broad range of temperature and stability preserved during storage.

## 6. Outlook

This paper deals with small pieces of cryogels into which laccase has been immobilized. It is obvious that the combination of the cryogel with the big pores, and the enzyme functions well regarding degrading of surplus dyes in wastewater from textile industry.

It should be mentioned that cryogels can be prepared in many different shapes. Monoliths and big discs could be created as filters for passing wastewater through. The pores are big enough to allow microorganisms to pass through the cryogel. What is fascinating is also the fact that there is very little backpressure when passing liquid through a cryogel. Furthermore, when treating wastewater with mixing, the cryogel can be protected from attrition by producing the gel within a plastic “housing” [53] . This latter prolongs the lifespan for the cryogels. These factors open up possibilities for scaling up treatment of industrial waters.
